# Using in vivo fluorescence lifetime imaging to detect HER2-positive tumors

**DOI:** 10.1186/s13550-018-0384-6

**Published:** 2018-04-04

**Authors:** Yasaman Ardeshirpour, Dan L. Sackett, Jay R. Knutson, Amir H. Gandjbakhche

**Affiliations:** 1 Section on Analytical and Functional Biophotonics, NICHD, NIH, Building 49, Room 5A82, Bethesda, MD 20892 USA; 2 Division of Basic and Translational Biophysics, NICHD, NIH, Building 9, Room 1E129, Bethesda, MD 20892 USA; 3 Laboratory of Advanced Microscopy and Biophotonics, NHLBI, Building 10, Room 5D14, Bethesda, MD 20892 USA

**Keywords:** Fluorescence lifetime imaging, Fluorescent biomarkers, Human epidermal growth factor 2 receptor, HER2 receptor, Affibody probe

## Abstract

**Background:**

Assessment of the status of tumor biomarkers in individual patients would facilitate personalizing treatment strategy, and continuous monitoring of those biomarkers and their binding process to the therapeutic drugs would provide a means for early evaluation of the efficacy of therapeutic intervention. Fluorescent probes can accumulate inside the tumor region due to the leakiness of its vascularization and this can make it difficult to distinguish if the measured fluorescence intensity is from probes bound to target receptors or just accumulated unbound probes inside the tumor. In this paper, we have studied the fluorescence lifetime as a means to distinguish bound HER2 specific affibody probes to HER2 receptors.

Our imaging system is a time-resolved fluorescence system using a Ti-Sapphire femtosecond pulse laser as source and Time correlated Single photon Counting (TCSPC) system as detector for calculating the lifetime of the contrast agent. HER2-specific Affibody (His6-ZHER2:GS-Cys) (Affibody, Stockholm, Sweden) conjugated with a Dylight750 fluorescent probe (Thermo-Fisher-Scientific, Waltham, Massachusetts) was used as contrast agent and six human cancer cell lines, BT-474, SKOV-3, NCI-N87, MDA-MB-361, MCF-7, and MDA-MB-468, known to express different levels of HER2/neu, are used in athymic mice xenografts.

**Results:**

By comparing the lifetime of unbound contrast agent (at the contralateral site) to the fluorescence lifetime at the tumor site, our results show that the fluorescence lifetime decreases as HER2 specific Affibody probes bind to the tumor receptors. A decrease of ~15% (100ps) in tumor fluorescence lifetime was observed in tumors with mid to high HER2 expression. Smaller decreases were observed in tumors with low-level of HER2 receptors and no change was observed in the non-HER2-expressing tumors.

**Conclusions:**

Using HER2-specific Affibody conjugated with the Dylight750 fluorescent probe as contrast agent, we demonstrated in live animals that change in fluorescence *lifetime* of the bound contrast agent can be used to assess the high to mid-level expression of HER2 expressing tumors in-vivo in only one measurement. The rationale is that the fluorescence lifetime of our specific probe is sensitive to affinity to, and specific interaction with, other molecules.

## Background

Efficacy of personalized cancer therapy, using specific drugs targeting tumor receptors depends strongly on characterization of tumor biomarkers and continuous monitoring of the tumor response to the targeted therapy. Due to the heterogeneity of tumors, biopsies may not be representative of all tumor regions [1]. In addition, during the therapeutic cycle, the number of times that a biopsy can be taken is limited. Alternative methods currently under consideration are based on pharmacokinetics of the targeted radionuclide or fluorescence probes after injection into the blood circulation. Extensive review of this line of research can be found in [2–6].

Development of Near InfraRed (NIR) fluorescent probes has significantly improved the capability of in vivo fluorescence imaging, due to low auto fluorescence background and deep penetration of NIR light in the tissue [7, 8]. Fluorescence imaging can be realized in the form of measuring the fluorescence intensity distributions and/or the fluorescence lifetime [9].

Fluorescence lifetime imaging is based on evaluation of the average time that electronically excited fluorophore stays in that excited state before its transition to a ground state, accompanied by photon emission in a longer wavelength [10]. As a result, long pass filtering can be used to eliminate the excitation light and increase dramatically the signal to noise ratio. It has been shown that fluorescence lifetime is largely independent of the concentration of the fluorophores and the intensity of the excitation light. On the other hand, it can be sensitive to local biochemical environment, especially direct molecular interactions. This property makes the fluorescence lifetime imaging a promising candidate for detecting and monitoring cancer-specific receptors in the diagnosis and treatment of diseases. It can also be used to investigate the effectiveness of early-phase treatment response by monitoring the binding of drug molecules to the tumor cells.

In this study, we targeted Human Epidermal Growth Factor 2 (HER2/neu) receptor, which is one of the important biomarkers in many cancers, including breast and ovarian cancer. Overexpression of this receptor is correlated with poor prognosis and resistance to specific chemotherapies [11, 12]. To optimize the treatment procedure, it is important to assess both the expression of the HER2 receptor in the diagnostic process and to monitor it in vivo over the course of treatment. To assess status of this receptor, we applied a HER2-specific Affibody conjugated to a near infrared (NIR) fluorescent dye. Affibody molecules are very stable proteins. They are relatively small (8.3 kDa), about 20 times smaller than antibodies [13]. Like many other small molecules, they can accumulate in the tumor through the leaky tumor vasculature. Their major diagnostic advantage is that they can be made highly specific to particular cancer cell receptors, for example, HER2. In addition, their specificity allows targeting of epitopes different from those targeted by standard therapeutic antibodies such as trastuzumab, making the concept of ‘image and treat’ possible [14]. The concept is that after the imaging probe binds to the specific epitopes of HER2 receptors, unattached probes will be washed out and mainly bound fluorescent ligands will then contribute to the signal from the tumor area at later times. This significantly improves the contrast of the tumor compared to the background.

Because of the leaky vascularization of the tumor and its enhanced permeability and retention (EPR) effect, using fluorescence intensity alone has limitations. It is difficult to establish whether the detected fluorescence intensity originates from the fluorescent agent bound to the specific cancer receptor or it comes simply from accumulated free fluorophores inside the tumor (due to EPR effect and leaky vascularization). Further, even though fluorescence intensity-based imaging methods have been successfully applied for analysis of some biomarkers in vivo [15, 16], this requires sequences of images at different time points to distinguish bound and unbound fluorophores, using pharmacokinetics of fluorescence intensity to take into account the accumulation and washout of the probe from blood circulation.

In several studies [17, 18], we were able to show the difference in fluorescence lifetime of targeted probes in tumors with high expression and no-expression of HER2 receptors. In our other study [14], we showed that the HER2 Affibody binds to a different epitope of the HER2 receptor than the therapeutic epitope targeted by such widely used monoclonal antibodies as trastuzumab or pertuzumab. This enables monitoring of HER2 expression during therapy without interference with the potential effect of these Ab-based drugs. In [17], we demonstrated in an animal model that the fluorescence lifetime can be used to monitor the efficacy of treatment with monoclonal antibodies like trastuzumab. These studies showed that our method can be used to monitor HER2 expression during therapy.

Here, we focus on whether our method can be used to characterize the level of expression of HER2, using six tumor models with different HER2 expression in live animals to evaluate the correlation of fluorescence lifetime with the expression of HER2 receptors in tumors. The results reveal significant differences between the fluorescence lifetimes of the tumor with high level (+3) and mid-level (+2) expression of HER2 receptor and the contralateral site. Small differences between the mean fluorescence lifetime of tumor and contralateral sites were observed in tumors with low-level (+1) expression of HER2 receptor. No fluorescence lifetime difference was observed in the tumors with no HER2 expression, where the optical probes have no affinity to the HER2 receptors; this shows that lifetime is relatively insensitive to EPR effects.

## Methods

In these experiments: HER2-specific Affibody (His6-ZHER2:GS-Cys) (Affibody, Stockholm, Sweden) conjugated with the Dylight750 fluorescent probe (Thermo-Fisher-Scientific, Waltham, MA) was used as HER2 targeted contrast agent with conjugation ratio of 1:1. Unit stoichiometry helps avoid any lifetime effects from heterogeneity or homotransfer.

Six human cancer cell lines, BT-474, SKOV-3, NCI-N87, MDA-MB-361, MCF-7, and MDA-MB-468 were obtained from the American Type Culture Collection (ATCC, Manassas, VA). The cells were grown in RPMI (BT-474, NCI-N87), DMEM-F12 (MDA-MB-468,) or DMEM (SKOV-3, MDA-MB-361, MCF-7) culture media supplemented with 10% fetal bovine serum (FBS) and 1% Pen/Strep (10,000 U penicillin, 10 mg/ml streptomycin) at 37 °C at 5% CO_2_ in a humidified environment. A solution of 0.05% trypsin and 0.02% EDTA (Invitrogen, Carlsbad, CA) in PBS was used for cells detachment.

Female athymic nude mice (*nu/nu* genotype, BALB/c background), 5 to 8 weeks old, were purchased from the Animal Production Program (NCI, Frederick, MD). This study was approved by the Animal Safety and Use Committee of NIH (Animal Study Proposal: ROB 117).

The tumors were initiated by subcutaneous injection of 5–8 million cells, suspended in 0.1 ml of 30% Matrigel solution (BD Biosciences, Bedford, MA), into the mouse right forelimb. Mouse forelimb location was selected to be far enough from kidney and renal track to ensure that their fluorescence signal would not affect the measured fluorescence signal from the tumor or its contralateral site. Growth of BT-474, MDA-MB-361, and MCF-7 cell lines was facilitated by subcutaneous implantation of estrogen pellets (0.72 mg, 90 days release, Innovative Research of America, Sarasota, FL) 24 h prior to cells injection.

Imaging studies were initiated when tumor diameter reached 0.5–0.8 cm. For fluorescence imaging, mice were anesthetized by inhalation of isoflurane (5% for induction, 2–2.5 for anesthesia maintenance, oxygen flow 1 l/min). Then, 10 μg of Affibody-DyLight-750 conjugate in 100 μl of PBS were injected intravenously. Near-Infra-Red optical imaging was performed using a previously described NIR fluorescence small animal imager [19]. The system is based on a time-domain technique, where an advanced time-correlated, single-photon counting device is used in conjunction with a high repetition-rate tunable femtosecond laser to detect timing of individual photons. The imager has a laser source for fluorescence excitation (λ = 750 nm), an emission filter (λ = 780 nm) for isolating fluorescence for detection, and scans in 2 mm steps in a raster pattern over the targeted area to produce two-dimensional images of the region of interest (ROI).

HER2-specific Affibody fluorescent probe was injected in 24 mice with 6 different tumor models, and the fluorescence lifetime of tumor and contralateral sites were measured in vivo. These cell lines include high level (+ 3) HER2-expressing human tumor carcinoma (BT-474, NCI-N87, and SKOV-3), medium level (+ 2) HER2-expressing human tumor carcinoma (MDA-MB-361), low level (+ 1) HER2-expressing human tumor carcinoma (MCF-7); and non-HER2-expressing human tumor carcinoma (MDA-MB-468). In all cases, HER2-specific Affibody (His6-ZHER2:GS-Cys) fluorescent probe was injected intravenously and imaging was performed 1 h after injection. We chose this time based on our measured fluorescence intensity at the tumor and contra-lateral sites at different time points for 5 h. About 1 h after injection, the fluorescent probe accumulation stabilizes at the tumor site and its intensity at the contra-lateral site is still considerably higher than background noise so it can be used to measure fluorescence lifetime of un-bound targeted agent.

To assess the fluorescence intensity at tumor and contra-lateral site, the mean of the fluorescence signal was obtained by averaging of the pixel values over the ROIs at the tumor and in normal tissue at the corresponding contralateral site. The fluorescence lifetime was estimated from a curve fitting of the data to a single exponential decay function. The tail of the time-resolved intensity data was truncated due to its greater susceptibility to photon transport path variations [17].

In our preclinical studies, the tumor was located in mouse forelimb subcutaneously; therefore, the depth of the tumor did not have a significant effect on the lifetime. However, for applications dealing with the deeply embedded tumors, the effect of the photon diffusion on the observed time-resolved fluorescence intensities should be taken into account [20].

To determine the HER2 protein level in the tumor tissue, an ELISA test was performed. Animals were sacrificed at the end of optical imaging followed by tumor tissue extraction. One part of the tumor was flash-frozen in liquid nitrogen and stored at − 80 °C. The HER2 level was measured using the c-ErbB2/c-Neu Rapid Format ELISA Kit (EMD Chemicals, Gibbstown, NJ), following the protocol provided by the manufacturer. Protein concentration in the tissue lysates was measured using BCA Protein Assay Kit (Pierce, Rockford, IL) according to manufacturer protocol. Data are expressed as nanogram of HER2 per milligram of tissue lysate ± SEM.

## Results

**Fig. 1:**
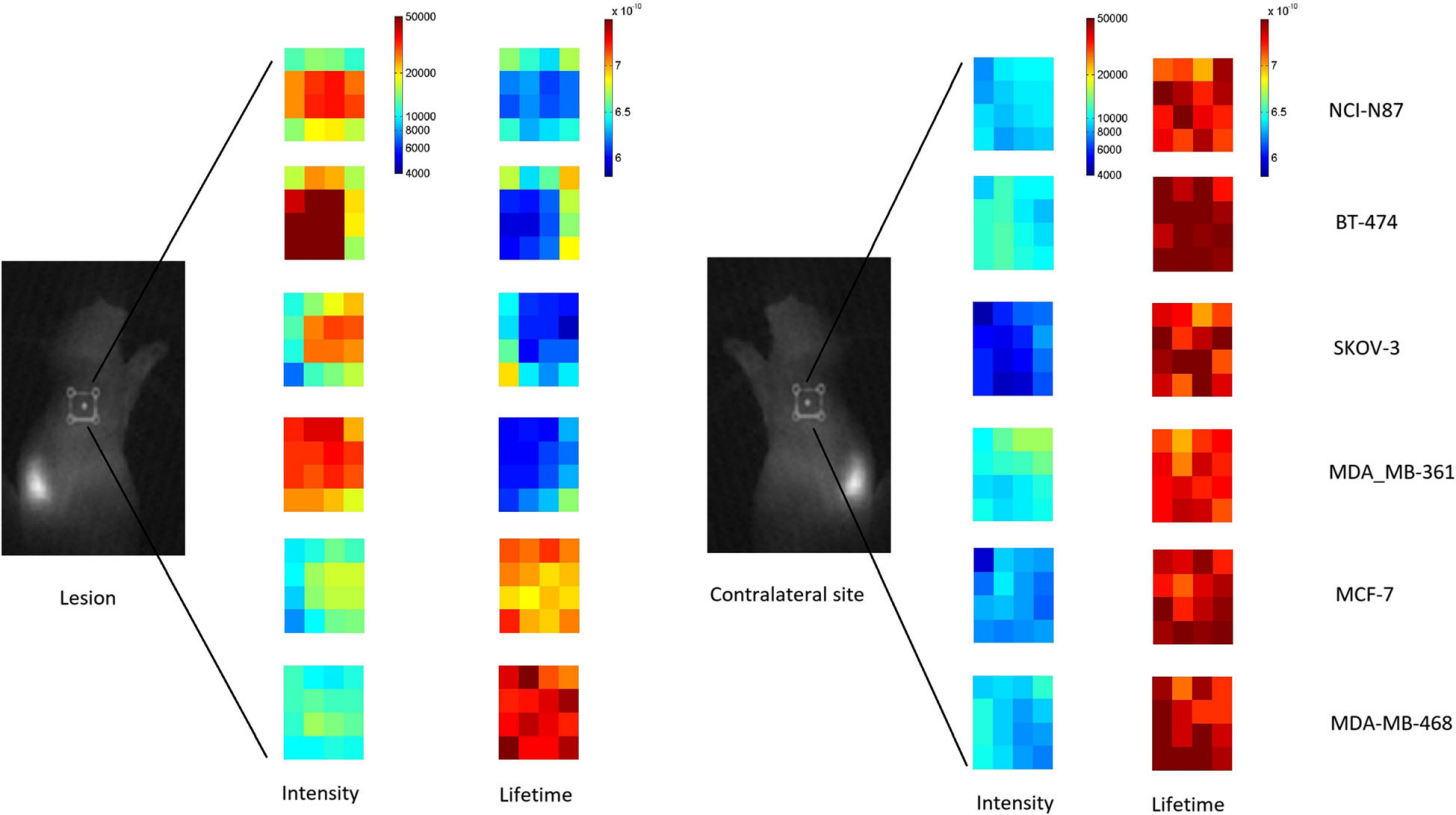
Fluorescence intensity (a.u.) and lifetime (sec) measured at the tumor and contralateral sites, 1 h after injection of HER2-specific Affibody probe

**Fig. 2:**
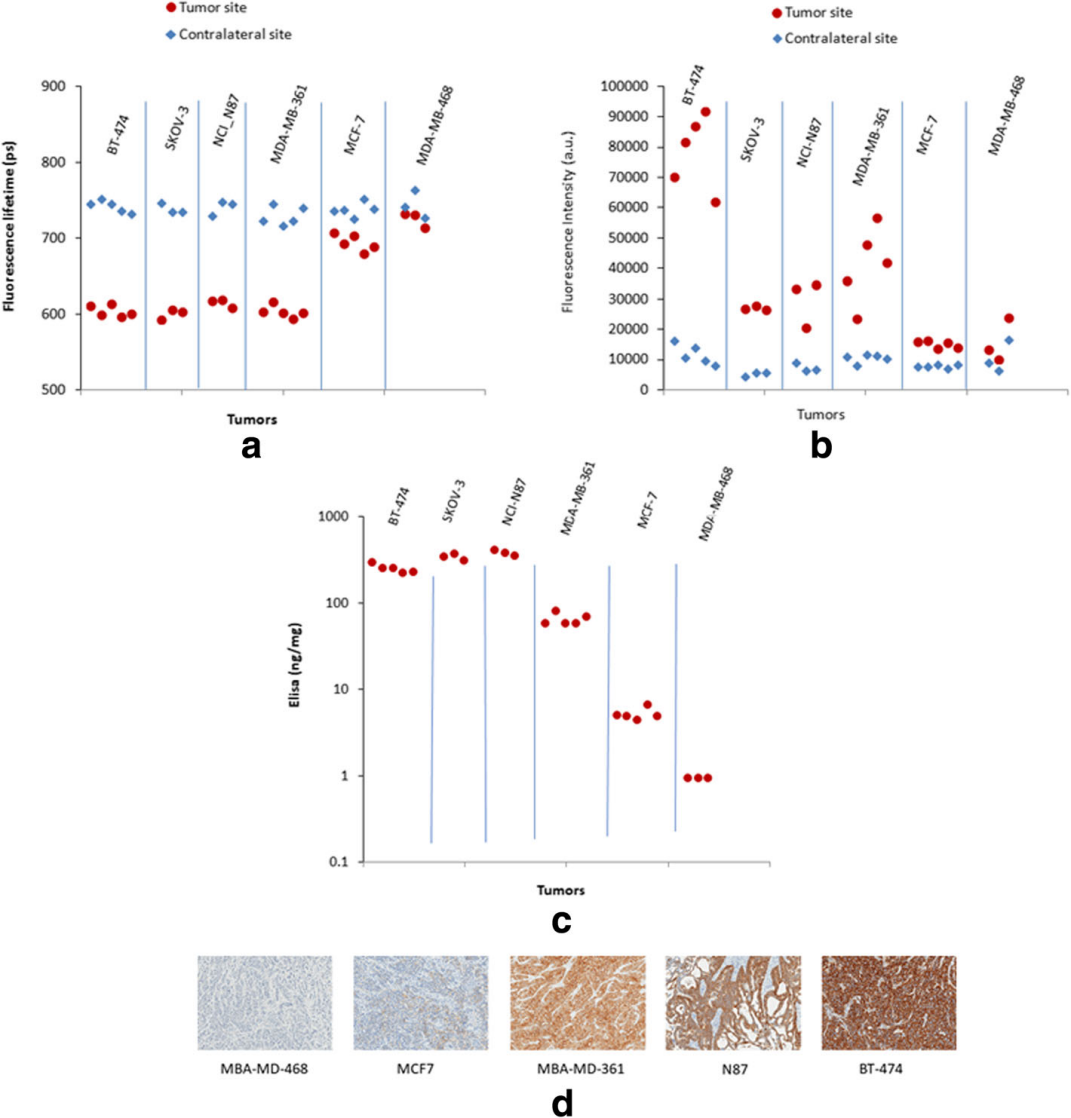
**a** Fluorescence lifetime and **b** fluorescence intensity measured in vivo at the tumor and contralateral sites, 1 h after injection of HER2-specific Affibody probe. **c** HER2 expression in each tumor measured by ELISA after tumor extraction. **d** Immunohistology images of five different tumor types with different HER2 expression, extracted 24 h after injection of HER2-specific affibody, and stained using DAKO HercepTest™ (Dako, Carpinteria, CA). Brown area shows HER2 positively stained cell membranes in the tumor tissue

Figure 1 shows the results of in vivo images of fluorescence intensity and lifetime at tumor and contralateral site, 1 h after injection of HER2-specific affibody probe. Figure 2 shows the mean value of fluorescence lifetime (Fig. 2a) and intensity (Fig. 2b) measured at the tumor area and the contralateral site, 1 h after injection.

Before injection, the fluorescence lifetime of Dylight 750, conjugated to HER2-specific Affibody (His6-ZHER2:GS-Cys), was measured as 0.71 ns. This lifetime was consistent with our in vivo measurements at the contralateral site.

**Fig. 3:**
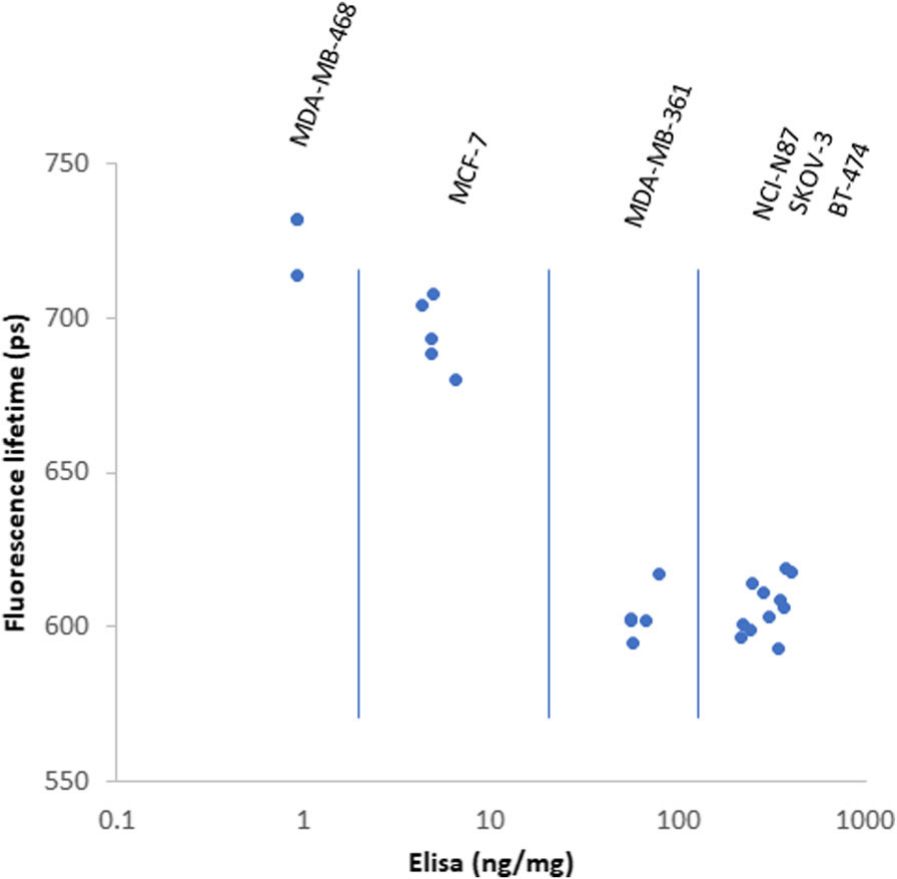
In vivo fluorescence lifetime at the tumor site vs. HER2 expression in the tumor measured by ELISA after tumor extraction

After imaging, the tumor was excised and its HER2 receptors were measured with ELISA. Figure 2c shows the HER2 expression in each tumor measured by ELISA. Figure 2d shows the immunohistochemistry images of each tumor type. Figure 3 shows the fluorescence lifetime at the tumor site vs. HER2 expression measured by ELISA.

These results show that the fluorescence lifetime of both high- and mid-level of HER2 expressing tumors was decreased significantly (~ 15% or ~ 100 ps) in the tumor area compared to the contralateral site 1 h after injection. Non-significant change was observed between the fluorescence lifetime of the tumor and contralateral sites in the non-expressing HER tumors (MDA-MB468). Small change between the mean fluorescence lifetime of the tumor and contralateral sites was observed in tumors with low-level of HER2 receptors perhaps due to the small ratio of bound to unbound receptors inside the tumor.

According to these results, the fluorescence lifetime can be used as a patient friendly approach for fast evaluation of tumors with mid- to high-level expression of HER2 receptors and can be a complement to our previous work for monitoring the treatment efficacy through only one measurement.

## Discussion

In this study, we investigated the fluorescence lifetime of HER2 targeting optical probe in six different cell lines with different level of HER2 expression.

Fluorescent probes can accumulate inside the tumor region due to the leakiness of its vascularization, and it would be difficult to distinguish if the measured fluorescence intensity is from probes bound to target receptors or simply from accumulated unbound probes inside the tumor. Therefore, comparison of the fluorescence intensity, originating from the tumor, with that of the contralateral site alone is not a good indicator of binding the fluorescent probe to the HER2 receptors. To quantify the binding of a fluorescent probe to a specific tumor receptor, determining the pharmacokinetics of the intensity contrast between tumor and contralateral site is required. This process can involve imaging of the tumor for up to several hours after a probe injection. From a practical point of view, the latter requirement may cause limitations for the clinical studies.

**Fig. 4:**
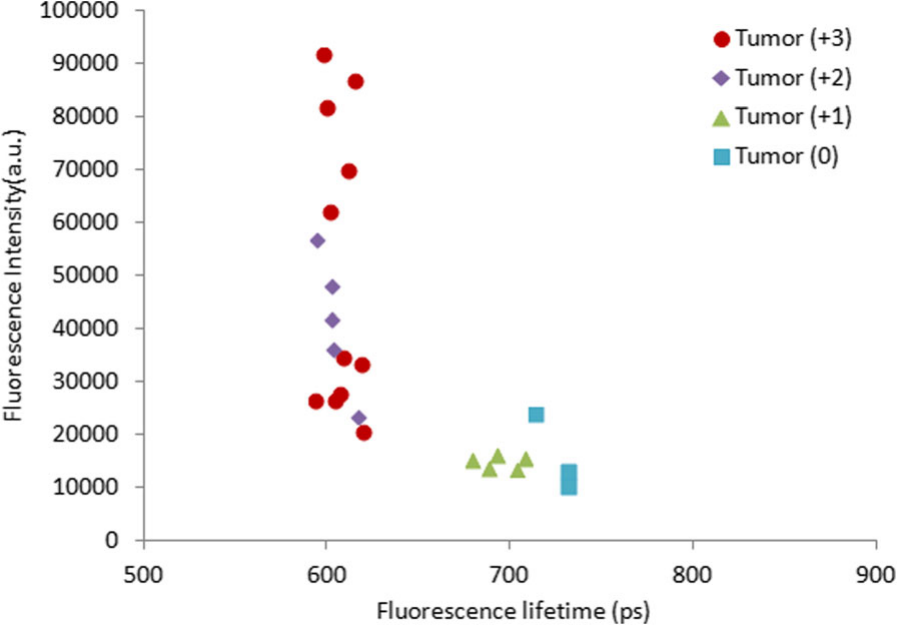
Fluorescence intensity vs. fluorescence lifetime of all tumors with high- (+ 3), mid- (+ 2), low- (+ 1) level and no (0) HER2 expression

On the other hand, fluorescence lifetime is neither sensitive to the intensity of excitation light nor concentration of fluorophores, and this is one of the main advantages of fluorescence lifetime over fluorescence intensity measurements (Fig. 4). Our results show a significant decrease in the fluorescence lifetime at the tumor site in both high- and mid-level of HER2 expressing tumors compared to the contralateral site. Non-significant change was observed between the fluorescence lifetime of the tumor and contralateral sites in the non-expressing HER tumors (MDA-MB-468). Small change between the fluorescence lifetime of the tumor and contralateral sites was observed in tumors with low-level HER2 receptors. According to these results, the fluorescence lifetime should be considered as a patient friendly approach for fast evaluation of tumors with mid- to high-level expression of HER2 receptors, using only one measurement in diagnostics and determination of efficacy of treatment intervention. In addition to the clinical applications, this method can also be considered as a potential laboratory tool in pre-clinical animal studies and pharmaceutical research.

## Conclusions

In this paper, we studied in live animal, the difference in the lifetime of HER2-specific Affibody conjugated with the Dylight 750 fluorescent probe bound to target receptors at the tumor site, to the lifetime of unbound probes (at the contralateral side) for six different human cell lines with different level of HER2 expression. Our results show that change in fluorescence lifetime of the bound contrast agent can be used to assess the high to mid-level expression of HER2 expressing tumors in vivo in only one measurement. The rationale is that the fluorescence lifetime of our specific probe is sensitive to affinity to, and specific interaction with, other molecules.
